# Attending Training Workshop of BLS/ACLS

**DOI:** 10.31729/jnma.7782

**Published:** 2022-10-31

**Authors:** Priya Yadav

**Affiliations:** 1 Nepalgunj Medical College and Teaching Hospital, Kohalpur, Banke, Nepal

**Keywords:** *cardiac arrest*, *cardiopulmonary resuscitation*, *life*, *medical students*, *workshops*

## Abstract

From the beginning of day one in medical school, students are always looking for ways to do something practical and skilled based instead of just reading a textbook. Having said that, learning a skill to save a life is the most fascinating thing. Although as a student, they may not have to attend to the patient, they could be present in accidents outside of the hospital, where they should be able to perform the necessary actions to save a life. After attending the training, one becomes somewhat confident that they can take control of the situation because they will know what to do until the emergency services arrive.

## INTRODUCTION

Cardiac arrest is the sudden cessation of cardiac function resulting in a respiratory and circulatory standstill.^1^ Thirty percent of acutely ill patients die before reaching the hospital in India and more than 80% of injured patients do not reach the hospital within the golden hour.^2^ Fear of injuring the victim, fear of poor performance and liability, reluctance to perform mouth-to-mouth cardiopulmonary resuscitation (CPR) for out-of-hospital cardiac arrest, early evacuation of a trauma victim, and stoppage of bleeding are the keys to this outcome.^2^ The average health personnel in our centre lack adequate knowledge in CPR/Basic life support (BLS) which should be addressed promptly. Since prior CPR training and clinical exposure influence the retention of knowledge, all healthcare professionals need to have some standard of CPR/BLS training and assessment.^3^ Health care professionals are expected to be competent to resuscitate from their first posting. In the United States, BLS training has been recommended for all healthcare professionals since 1966.^4^

## WHY ATTEND LIFE SUPPORT TRAINING?

Cardiac arrest is a significant health risk with approximately 356,000 people suffering a sudden cardiac arrest (SCA) per year in the United States (US).^5^ Due to the high morbidity and mortality of cardiac arrest, CPR training has been included in emergency care training for medical students throughout the US.^6^

Attending such training is not a requirement for graduation. Students can complete their education in medical school even if they attend life support training. But when we think about all the benefits someone can gain from attending such a workshop, it's hard to ignore that those who do not attend conferences are missing out. The medical curriculum is designed to teach the theory and practice of medicine and because of this, lectures and tutorials can only provide scenarios and challenges we will face in healthcare. But training like this opens our eyes to the realities of clinical practice and helps us to understand how health decisions are made, and what future health challenges await us. This can be difficult to cover in the classroom but it is one of the most important and valuable experiences for our future practice.

Anyone can learn to perform basic CPR by watching a video at home, but it's the comprehensive training and certification process that gives healthcare providers the courage to use their skills in an emergency. That's because training is not passive. CPR training gives students the confidence to act in stressful situations. Performing CPR is a dynamic process. It requires not only hands-on know-how but also the ability to delegate tasks and manage the type of high-pressure situations that don't happen every day, even in health care. Being able to practice CPR skills in a classroom environment gives healthcare providers an edge and helps them be effective leaders when the time comes. In any healthcare environment, advanced CPR certifications are valued. They demonstrate an applicant's personal initiative and sense of responsibility.^7^ Once you have trained in Advanced Cardiovascular Life Support (ACLS)/BLS you will have acquired a skill that you can use for the whole of your life. Since this training provide us with an important skill so it makes good sense to get trained as soon as you can.

## EXPERIENCE AS A MEDICAL STUDENT

Medical students are always in the phase of learning and trying to learn the skills required while treating patients. For this, they are exposed to different trainings, seminars and workshops. On that note, opportunities to attend the ACLS/BLS training workshops are available for medical students in certain parts of our country as well. However, such trainings can be overwhelming yet exciting for medical students.

Certain workshops use questionnaires for pretest evaluation. The workshops usually cover the importance of BLS/ACLS, techniques and ways to promptly recognize cardiac arrest, give high-quality chest compressions, and deliver appropriate ventilation with both theortical and practical sessions. Dummies are usually provided by the instituion in order to ensure that each and every participant gets a hands on experience. The procedures can become tiring and difficult for some students at first but the excitement and satisfaction that follows can be worth an experience.

The workshops can also cover drugs used in ACLS and the emergency room, introduction to a defibrillator, with practical session on the usage and techniques of operation. The worshops can be followed by post tests which if satisfactory, the participants are provided with a certificate which can be a great boost for their medical career.

That being said as a medical student, attending BLS/ACLS training and being certified is a rewarding experience. The training can indeed be an amazing experience, and the students can get to learn a lot from a brief interaction. An additional benefit of such trainings can be the learning to remain composed and respond appropriately when leading the code team and deciding the best possible way to try and save a patient's life.

## WAY FORWARD

The question of what makes a good doctor is not easy to answer. But, there are some skills that doctors must have and must acquire throughout their days of training to become better physicians. Likewise learning BLS/ACLS qualifies you to step in and make a difference in case someone needs serious help and prevent serious injuries to others around them at home, work, and in public. In a cardiac arrest emergency, every second counts. If performed immediately, CPR can double or triple a person's chance of survival. Empowering all youth with CPR and AED (automated external defibrillator) training will dramatically increase the number of first responders in communities each year and save lives.
